# Review

**Published:** 1839-09

**Authors:** Chapin A. Harris

**Affiliations:** Baltimore.


### REVIEW.

[ BY CHAPIN A. HARRIS, M. D. OF BALTIMORE. ]

Dentologia, a poem, on the diseases of the teeth, and their proper remedies, by Solyman Brown, A. M.; with notes, practical, historical, illustrative, and explanatory, by Eleazar Parmly, Dentist.—New-York, Peabody & Co. 1833.

We have seldom enjoyed a richer treat than has been afforded us by the perusal of the above work; and believing that there are many who have not yet seen it, we would not only commend it to the attention of our professional brethren; but also, to the notice of all who feel any interest in the preservation of the teeth. We saw in several Journals of the day, soon after its publication, very flattering notices of it, but not being able to procure a copy at that time, we were deprived of the pleasure which we have more recently enjoyed, of a perusal of its pages. From the extracts which we then saw, we were convinced that it was a work of considerable merit; but had no idea that it contained so great a variety of interesting information on the subject of dentistry. The author not only evinces a thorough and scientific knowledge of all the principles of the art, but also a mind highly cultivated and richly imbued with poetic fancy. He exhibits the science in its most fascinating colors—presenting the dry details of its general principles in the glowing and eloquent language of a rich poetic imagination; and judging of the capabilities of Mr. Brown as a practitioner, from this production, we believe we hazard little in saying that he is destined soon to occupy, if he does not already, a distinguished place in the profession. The ardent love which he has here manifested for it, will not allow him to rest satisfied with any thing short of the highest excellence; and well would it be for those who are so unfortunate as to need its aid, were all who have embraced it, actuated by a similar zeal and the same high-minded and praise-worthy motives. Were this the case, it would have an importance in public estimation, more commensurate with its value, than that usually attached to it.

The art of the Dentist is thus introduced.

"If nature thus, instructive, deigns to trace
The soul in every feature of the face;
If lovely virtue there displays her charm,
And guilty passions ring the loud alarm;
Arouse, thou slumbering fair! and learn to see
That heaven commits thy destiny to thee.
Is virtuous love thy aim? deserve the prize:
Or friendship? Know that here the secret lies:—
To be—and to appear what men approve:
Their friendship thus is won—and thus their love.
Be mine the pride in measured verse to raise
A plain, but lasting monument of praise,
To that distinguished science, known of yore,
Designed departed beauty to restore.—
The Dental Art, by Greece and Rome admired,
When women to imperial thrones aspired;—

Those mighty states were both to ruin hurled,
But lo! their art survives to bless the world."

On the difficulties of entering into the details of a science in verse, the author makes the following allusion.

"Full well I know 'tis difficult to chime
The laws of science with the rules of rhyme;
Plain, vulgar prose, my subject seems to claim,
Did not ambition prompt the higher aim,
The nobler pride, by more laborious care,
To speak in numbers that shall please the fair."

Of the abuses of the art, Mr. B. seems fully aware, and judiciously cautions those seeking its aid against ignorant pretenders. He says,

"Beware of those whom science never taught
The hard, but useful drudgery of thought,
* * * *
On just desert, let all success attend,
And patient merit never want a friend."

That it has suffered much from the ignorant and unskilful manner in which its duties have been but too frequently exercised, is a lamentable truth; but we are cheered with the belief, notwithstanding the contrary is often now the fact, that the time is not far distant when more correct notions on the subject will be entertained; and when only such as are qualified to practice it, will be permitted to do so.

The following justly deserved and highly complimentary tribute is addressed to the gentleman to whom this poem was presented, Mr. E. Parmly.

"To thee, companion of my happiest days,
The general voice awards superior praise;
'Twas nobly won, by sacrifice of ease,
'Mid raging tempests and through stormy seas."

First and second dentition form the subject of the second canto, and on the importance of proper attention to the former, we extract the following.

"Be prompt to act:—'tis dangerous to delay,
Since life awaits the issue of a day:—
Reject the gentler means:—employ the best:—
Let unobstructed nature do the rest.
This rule neglected, many a smiling form,
With beauty bright, and life blood glowing warm,
Its parents' pride, a floweret in its bloom,
Descends lamented to an early tomb.

Was this advice more generally attended to, the mortality among infants would be greatly diminished, and the mother would be much less frequently called upon to mourn the early loss of her dearly cherished offspring. The fragile and irritable state of the system at the early age at which this operation of nature is performed, is such as to be easily affected, and there can be but little doubt but that most of the diseases met with in infancy, are attributed to the irritation produced by the cutting of the primative teeth. The great relief often afforded by the lancing of the gums, would, at least, seem to render this opinion highly probable.

On the effects liable to result from inattention to the first and second sets of teeth, he observes ;

> " Nor less the danger when the first array—
> The infant teeth—alternately decay,
> Or yield succession to a hardier race
> With marked reluctance ; for, in either case,
> Neglect will bring repentance in its train ;
> In one deformity ;—the other pain,
> Or fell disease :—but timely care may still
> Avoid the danger, or repair the ill.
> If pains ensue, and neighboring parts inflame,
> Extraction is the cure ; and 'tis the same
> If nature's law, obstructed in its course,
> Should meet resistance from opposing force :
> For this resisting force, howe'er remote,
> Meets in the dental art its antidote ;
> Pain flies its presence ; anguish wipes her tear ;
> To hope's fond vision rain-bow hues appear ;
> Pale, trembling beauty hushes her alarms,
> And beaux, admiring, own her added charms.
>
> Now mark the contrast, in some hideous face,
> Robbed by neglect, of symmetry and grace :—
> Behold those organs, formed on nature's plan,
> To serve important purposes to man ;
> * * * *
> Deranged, displaced, distorted, set awry,
> Disgusting objects of deformity."

After adverting to the effects produced upon the countenance, especially that of the female, by an irregularly arranged, and badly formed denture, and to the resources of the dentist, he says ;

> " Such benefits this useful science lends
> To earliest youth ;—and yet its aid extends
> To following years, assuaging mortal pain,
> And oft restoring beauty's flowery reign."

Passing on to the third canto, he commences with an apostrophe to luxury,—speaks of its effects upon the general health of the system, and upon the teeth ; also of gangrene and other diseases, and as an example, quotes the fate of Urilla; and concludes with the description of caries, its effects and tooth-ache. The whole of this canto is very interesting, and we regret that we can give but a few short extracts. We commence with two on luxury.

Of all the ills that ante-date the doom
Of erring mortals, and erect the tomb
So near the cradle, shortening to a span
The fleeting life of transitory man,
The worst is luxury :—Infrequent flies
The lightning's fatal bolt; the lowering skies
Are seldom darkened by the whirlwind's wrath,
Or loud tornado's devastating path.
Beneath the ocean wave though some expire,
And others by the fierce volcano's fire ;
Though savage war can boast his thousands slain,
On tented field, or bosom of the main ;
Yet few the victims of these fates malign,
Compared, intemperate luxury ! with thine."

After finishing the description of the fate of Urilla, he gives the following caution to others :

" Let every fair one shun Urilla's fate,
And wake to action e'er it be too late ;—
Let each successive day unfailing bring
The brush, the dentifrice, and from the spring,
The cleansing flood :—the labour will be small,
And blooming health will soon reward it all.
Or, if her past neglect preclude relief,
By gentle means like these, assuage her grief ;
The dental art can remedy the ill,
Restore her hopes, and make her lovely still.
Yet other evils may her care engage,
The offspring of an epicurean age.
Destructive caries comes with secret stealth
T' avenge the violated laws of health :
Dilapidates the teeth by slow decay,
And bears them all successively away.

The treatment of the diseases of the teeth—the supplying their loss with artificial, and an " eulogium on those who labour for the good of mankind," are the subjects treated of in the fourth canto. The author commences by invoking the aid of the dentist's art. He says,

" Auspicious art ! before whose magic spell,
Disease and pain shrink shuddering back to hell,
Whose touch, like that misterious gem of old,
That changed all baser metals into gold,
Restores the faded flow'ret to its bloom,
And saves the victim from the threatening tomb :—
Direct my song, and teach me to rehearse
In the smooth numbers of enchanting verse,
Those various stratagems employed by thee,
To soothe the pangs of frail humanity.

The subject chosen by Mr. Brown for his poem is a difficult one, affording, as must be acknowledged, comparatively little scope to the imagination, yet it cannot but be admitted that he has been eminently successful. " Dentologia" is a rich gem in the literature of Dentistry, and should be possessed by every member of the profession.

